# *Hepacivirus* Infection in Domestic Horses, Brazil, 2011–2013

**DOI:** 10.3201/eid2012.140603

**Published:** 2014-12

**Authors:** Bernard Salame Gemaque, Alex Junior Souza de Souza, Manoel do Carmo Pereira Soares, Andreza Pinheiro Malheiros, Andrea Lima Silva, Max Moreira Alves, Michele Soares Gomes-Gouvêa, João Renato Rebello Pinho, Heriberto Ferreira de Figueiredo, Djacy Barbosa Ribeiro, Jonan Souza da Silva, Leopoldo Augusto Moraes, Ana Silvia Sardinha Ribeiro, Washington Luiz Assunção Pereira

**Affiliations:** Universidade Federal Rural da Amazônia, Belém, Brazil (B.S. Gemaque, H.F. de Figueiredo, D.B. Ribeiro, J.S. da Silva, A.S.S. Ribeiro, W.L.A. Pereira);; Instituto Evandro Chagas, Belém (B.S. Gemaque, A.J.S. de Souza, M.C.P. Soares, A.P. Malheiros, A.L. Silva, M.M. Alves);; Universidade de São Paulo, São Paulo, Brazil (A.J.S. de Souza, M.S. Gomes-Gouvêa, J.R.R. Pinho);; Universidade Federal do Pará, Belém (L.A. Moraes)

**Keywords:** Hepatitis C virus, *Hepacivirus*, horse, Latin America, viruses

**To the Editor**: An estimated ≈150 million persons (3% of the world population) are chronically infected with hepatitis C virus (HCV). This virus is the prototype of the genus *Hepacivirus* and a major cause of liver cirrhosis and hepatocellular carcinoma around the world. Every year, 3–4 million persons become infected with HCV, and ≈350,000 die of this infection (*1*).

Development of an HCV vaccine has been hampered by the difficulty of in vitro cultivation of the agent and by lack of animal models for studies of viral functions and host immune reactions (*1*,*2*). The only effective experimental model that can be infected with HCV and in which the course of infection is similar to that in humans is the chimpanzee.

Since the discovery of HCV-like virus in dogs with respiratory disease and nonspecific gastrointestinal disorder in the United States in 2011, tentatively named canine hepacivirus (*2*), new hepaciviruses have been detected in insectivorous bats (*3*), Old World monkeys (*4*), wild rodents (*5**,**6*), and domestic horses (*6**–**8*). These animals could potentially serve as HCV models, but the accuracy of HCV tissue tropism, pathology, and immunology in natural hosts needs to be demonstrated. 

No official nomenclature for these recently described hepaciviruses has been defined by the International Committee of Viral Taxonomy. In this article, we refer to the viruses detected in horses as conventional nonprimate hepaciviruses (NPHVs). The aim of this study was to verify NPHV infection in horses from 8 locations in the eastern Brazilian Amazon.

During January 2011–November 2013, serum samples were collected from 300 equids in 7 cities and 1 district of the State of Pará, Brazil (Technical Appendix). Samples came from 265 horses (*Equus caballus*), 30 mules (*Equus mulus*), and 5 donkeys (*Equus asinus*). All procedures for obtaining and using the samples were approved by the Ethics Committee on the Use of Animals in Research from the Evandro Chagas Institute (protocol code 0023/2012 CEUA/IEC/CENP/SVS/MS).

Viral RNA was extracted from serum samples by using the TRIzol LS Reagent (Invitrogen, Carlsbad, CA, USA), and a target sequence in the nonstructural 3 protein (NS3) region of the NPHV genome was amplified from the cDNA by using a previously reported nested PCR protocol (*8*). Second-round product reactions of ≈380 bp were considered positive.

We used the BigDye Terminator version 3.1 Cycle Sequencing Kit and an ABI 3500 Genetic Analyzer (both from Applied Biosystems, Foster City, CA, USA) for sequencing. To obtain consensus sequences, we used BioEdit software, version 7.0.5.3 (http://www.mbio.ncsu.edu/BioEdit/BioEdit.html). To phylogenetically analyze the virus sequences based on an NS3 partial nucleotide sequence of 294 bp, we used the maximum-likelihood method with the Kimura 2-parameter model in MEGA version 5.1 (http://www.megasoftware.net) (bootstrap, 1,000 replicates). The nucleotide distance was calculated by using PAUP version 4.0 software (http://paup.csit.fsu.edu/). The sequences obtained in this study have been assigned GenBank accession nos. KJ469442–KJ469466.

Of the 300 serum samples, 25 (8.3%) were positive for NPHV by nested reverse transcription PCR; these results were confirmed by nucleotide sequencing. This prevalence of NPHV infection among horses in Brazil is higher than that reported for other countries (*6**–**8*).

The prevalence among competition horses (19.1%) was remarkably higher than that among farm (6.3%) and cart horses (6.2%). Many possibilities for the higher prevalence in the first group, such as exposure to nonsterile needles shared by a large number of animals, must be considered; however, further studies are needed to investigate this hypothesis.

No mules or donkeys tested in this study were infected by NPHV. A similar finding for another study has been reported (*8*), which might suggest that these species are not natural hosts for NPHV.

Until now, it has been impossible to associate *Hepacivirus* infection in horses with clinical hepatic disease (*7**,**8*). All infected horses for which information was available were healthy at the time of sampling. Our finding of high NPHV prevalence among horses is consistent with findings of previous studies in which NPHV-mediated disease was not found, suggesting that the course of infection is chronic and largely asymptomatic, similar to that of HCV infection in humans.

The sequences reported in this study shared close genetic relationships with previously reported NPHV sequences from dogs and horses (Figure) (*2*,*6**–**8*). The nucleotide distances of the sequences ranged from 0 to 19.9%.

**Figure:**
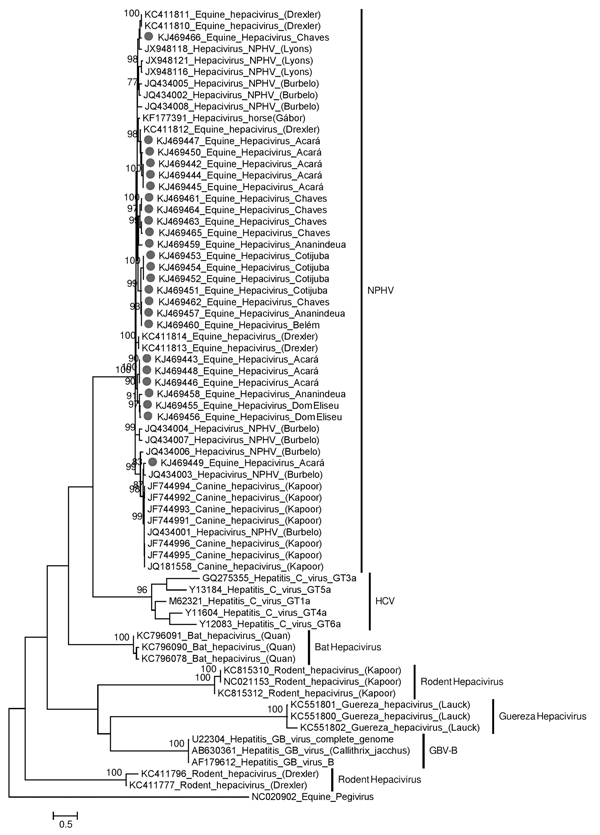
Maximum-likelihood phylogenetic tree of the partial nucleotide sequences of the nonstructural protein 3 region (294 bp) of *Hepacivirus*. The retrieved sequences from GenBank are indicated by the accession number followed by the species from which each was isolated. The 25 sequences obtained from 300 equids in 7 cities and 1 district of the State of Pará, Brazil, during January 2011–November 2013, are indicated with a dot and are identified by their GenBank accession numbers followed by their community of origin. Bootstrap values ​​(1,000 replicates) >70% are listed at the nodes. One sequence of equine *Pegivirus* was used as an outgroup. GBV-B, GB virus B; HCV, hepatitis C virus; NPHV, nonprimate hepaciviruses. Scale bar indicates nucleotide substitutions per site.

Many of these new isolates were grouped according to geographic origin, except for 2 sequences that grouped distantly from other isolates from Neotropical regions. One isolate (KJ469449), from a Quarter horse, was closely related to NPHV detected in horses in the United States (*7*); similarity was ≈94%. The other sequence from a Neotropical region (KJ469466), from a Marajoara horse, grouped closer to the NPHV from horses in Germany (*6*); similarity was as high as 95%.

NPHV, one of the viruses most closely genetically related to HCV, is present in the Neotropics and was identified in the eastern Brazilian Amazon. The infection seems to be enzootic in the studied horse population and to be geographically dispersed in northeastern Pará State.

Technical Appendix Locations in the State of Pará (eastern Brazilian Amazon), Brazil, where blood samples were collected from 300 equids for nonprimate hepacivirus testing.
