# Supplementary material for: Hepacivirus Infection in Domestic Horses, Brazil, 2011–2013
Source: Emerg Infect Dis. 2014 Dec;20(12):2180–2. doi: 10.3201/eid2012.140603 (PMC4257787; doi:10.3201/eid2012.140603)
Supplement: Technical Appendix — Locations in the State of Pará (eastern Brazilian Amazon), Brazil, where blood samples were collected from 300 equids for nonprimate hepacivirus testing. [file 14-0603-Techapp-s1.pdf]

DOI: http://dx.doi.org/10.3201/eid2012.140603

Hepacivirus Infection in Domestic Horses, Brazil, 2011–2013

Technical Appendix

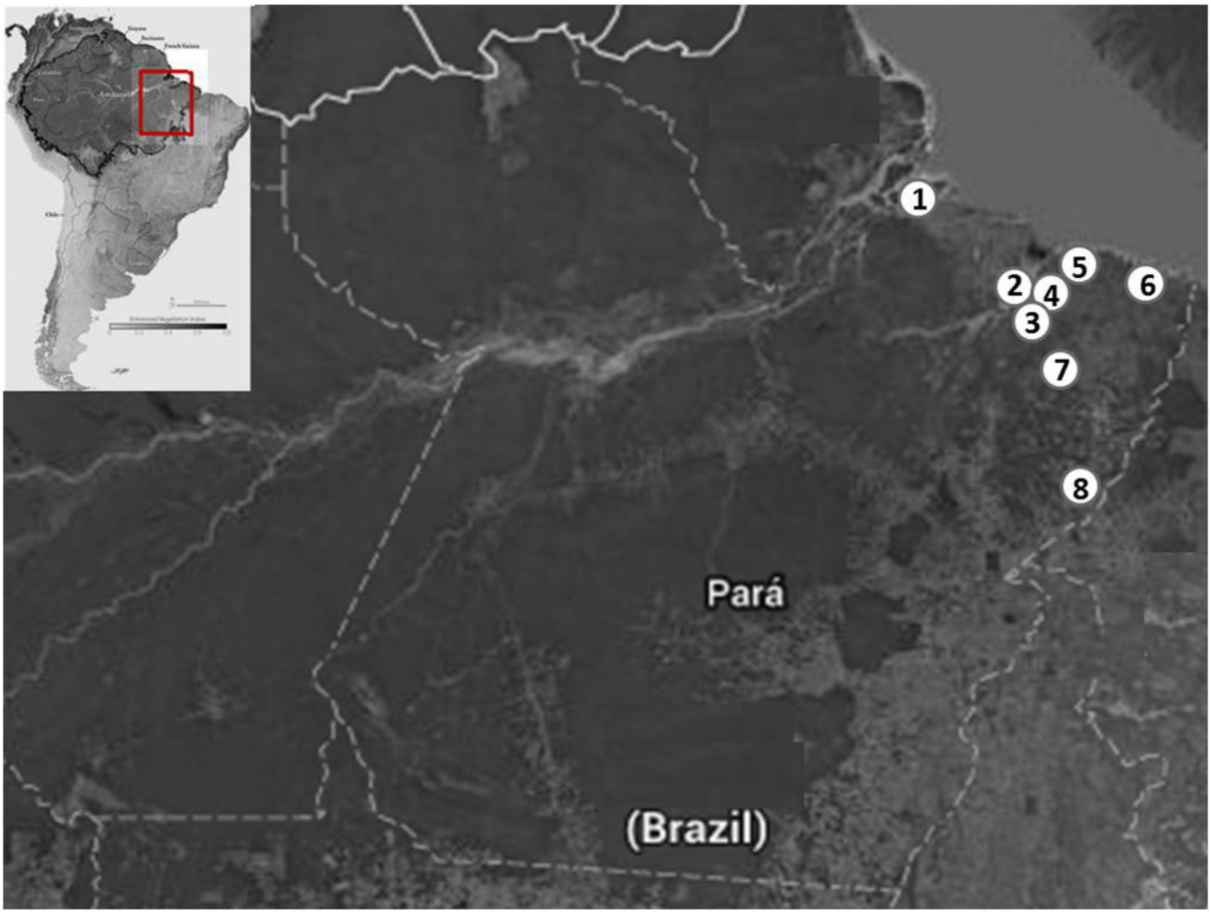

Technical Appendix Figure. Locations in the State of Pará (eastern Brazilian Amazon), Brazil, where blood samples were collected from 300 equids for nonprimate hepacivirus testing. The numbers correspond to municipalities, followed by latitude and longitude. 1, Chaves (00°09′36″S; 49°59′18″W); 2, Cotijuba (01°13′04″S; 48°32′44″W); 3, Belém (01°27′21″S; 48°30′16″W); 4, Ananindeua (01°21′56″S; 48°22′20″W); 5, Santo Antonio do Tauá (01°09′07″S; 48°07′46″W); 6, Peixe-boi (01°11′31″S; 47°18′44″W); 7, Acará (01°57′39″S; 48°11′48″W); 8, Dom Eliseu (04°17′06″S; 47°30′18″W).
